# Damage Detection of Common Timber Connections Using Piezoceramic Transducers and Active Sensing

**DOI:** 10.3390/s19112486

**Published:** 2019-05-31

**Authors:** Fang Han, Jinwei Jiang, Kai Xu, Ning Wang

**Affiliations:** 1 College of Science, Wuhan University of Science and Technology, Wuhan 430065, China; hanfang@wust.edu.cn; 2 Department of Mechanical Engineering, University of Houston, Houston, TX 77204, USA; jjiang7@uh.edu; 3 College of Urban Construction, Wuhan University of Science and Technology, Wuhan 430065, China; xukai@wust.edu.cn

**Keywords:** timber connection damage monitoring, piezoceramic transducer, lead zirconate titanate (PZT) transducers, active sensing method, wavelet packet-based energy analysis

## Abstract

Timber structures have been widely used due to their low-cost and environmental-friendly properties. It is essential to monitor connection damage to ensure the stability and safety of entire timber structures since timber connection damage may induce catastrophic incidents if not detected in a timely manner. However, the current investigations on timber connections focus on mechanical properties and failure modes, and the damage detection of timber connection receives rare attention. Therefore, in this paper, we investigate the damage detection of four common timber connections (i.e., the screw connection, the bolt connection, the decussation connection, and the tooth plate connection) by using the active sensing method. The active sensing method was implemented by using a pair of lead zirconate titanate (PZT) transducers: one PZT patch is used as an actuator to generate stress waves, and the other works as a sensor to detect stress waves after propagating across the timber connection. Based on the wavelet packet energy analysis, the signal energy levels of received stress waves under different damage extent are quantified. Finally, by comparing the signal energy between the intact status and the damage status of the timber connection, we find that the energy attenuates with increasing severity of the connection damage. The experimental results demonstrate that the active sensing method can realize real-time monitoring of timber connection damage, which can guide further investigations.

## 1. Introduction

Timber structures have a long history of use globally [1] since timber is a ubiquitous and environmental-friendly material that satisfies the requirements for sustainable development. Generally, to construct large-scale structures, individual timber elements are held together through different types of connections. However, the deformation and shrinkage of timber members caused by various issues (i.e., excessive load, wind vibration, or earthquake) can lead to damage to timber connections over time. Prior investigation has demonstrated that the stability and safety of entire timber structures greatly depend on the integrity of connections [2], and the insufficient integrity of connections is the major cause of failures in timber structures [3]. In addition, some researchers noticed that the peak stress, rotational stiffness, and bearing capacity of joints deteriorated due to damages of timber connections [4]. Thus, the monitoring of timber connection damage holds importance in timber and construction industries.

Nowadays, the most commonly used timber connections [5,6,7,8,9] in industries include the mortise–tenon joint, connections through screws, bolts, nails, dowels, tooth plates, rings, and glued connections. With the development of timber constructions [10], the investigations and optimal design of timber connection have received increasing attention over the past decades [11]. First, the mechanical properties [12,13,14] and the failure modes of timber connections have been studied by using theoretical analysis and numerical simulation. For instance, Robert et al. [15] researched the brittle and ductile failure modes of lateral timber–steel–timber connections, which were fastened by metal dowel-type joints, to estimate carrying capacity of timber connections. Analytically, Jorge et al. [16] extracted the key factors that influenced the failure modes of different timber joints, namely geometrical configurations and mechanical properties of joints. Similarly, by using numerical simulation, Daniel et al. [17] analyzed the brittle failure behavior of double shear connections in timber structures. In addition to the above analytical and numerical methods, experimental researches [18,19,20] were also conducted to study the effect of damages on the strength of different timber connections. Typically, Chen et al. [21] developed two pseudo-static tests on a full-scale specimen of a timber building, to obtain degradation of the bearing capacity of traditional mortise–tenon connections under different vertical loads. Pouyan et al. [22] predicted the ultimate capacity and failure mode of timber connections accurately, by analyzing data from a timber connection laboratory. Second, some researchers carried out investigations on the optimal design of timber connections. According to the Eurocode 5 standard, Carmen et al. [23] established a comprehensive database that contained test results on timber connections held by ringed shank nails to obtain reliable parameters for designing nailed joints. Based on the mixed integer nonlinear programming approach, Silih et al. [24] optimized the shape and discrete sizing of timber trusses. However, it is worth noting that the investigation on damage monitoring of timber connections is limited.

In recent years, the structural health monitoring (SHM) methods [25,26,27] based on smart materials have been actively researched in different fields. Particularly, the lead zirconate titanate (PZT) is suitable for SHM [28,29], since it has broad-band frequency response, easy-to-implement, the dual functions of sensing and actuating, and good durability [30,31]. For these reasons, PZT transducers are often used to generate and detect stress waves and ultrasonic waves in SHM and damage detection [32,33,34]. Moreover, the PZT can be fabricated into various shapes to satisfy the special requirements across multiple industries. However, it is worth noting that the biocompatibility of the PZT is unsatisfactory, since it consists of the lead element, which is harmful to the environment. Therefore, more advanced piezoelectric materials [35,36] will be considered in future investigations. Another issue that the PZT material faces is environmental temperature variation. Prior investigation [37] has proven that temperature variation has a significant effect on monitoring results based on PZT transducers, and several compensate methods, including the effective frequency shift (EFS) algorithm and the artificial neural network (ANN), have been proposed to address this issue. Amongst various SHM methods based on PZT transducers, the active sensing method is a common approach, due to its effectiveness in damage detection, particularly connection looseness. For instance, Wang et al. [38,39] applied the active sensing method to quantify the bolt looseness accurately. Moreover, in terms of timber damages, Zhang et al. [40] applied the active sensing method and a wavelet-packet damage index to detect holes and cracks in timber structures. Zhang et al. [41] demonstrated that the PZT-enabled active sensing method could monitor the moisture content of timber specimens in real-time. Zhao et al. [42] monitored the bolt connection of a timber structure based on the time-reversal method and PZT transducers. However, the investigation on damage monitoring of different types of timber connections using the active sensing method has not been reported, to the best knowledge of the authors.

In this paper, the investigation on damage detection of four common timber connections was conducted by using the active sensing method, and PZT transducers were used to enable active sensing. For each connection specimen, one PZT patch served as an actuator, and the other worked as a sensor. Stress waves generated by the actuator propagated through the timber connection, and they were received by the sensor. Since the dissipation of stress waves is highly sensitive to the defects along the propagation path, the signal energy decreases significantly with damage expansion. Then, to quantify the damage extent, a wavelet packet energy index was applied to compute signal energy of received stress waves. The results indicate that the active sensing method can estimate the damages of different timber connections efficiently.

## 2. Principles

### 2.1. Active Sensing

**Figure 1:**
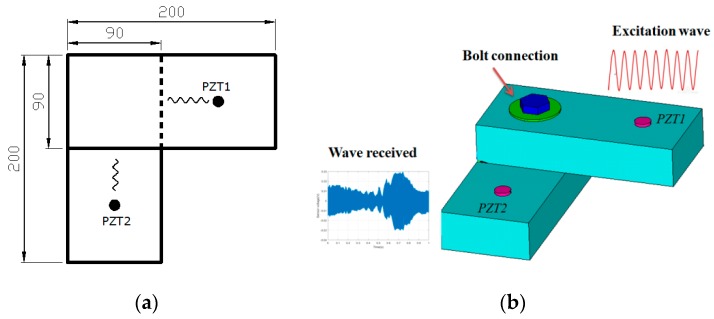
Schematic of active sensing method for damage monitoring of timber connection: (**a**)The dimension of the timber specimen (unit: mm); (**b**) the stress wave propagation diagram. PZT: lead zirconate titanate.

In this paper, the active sensing method based on PZT transducers is used to detect the damage of different types of timber connections, and the schematic is depicted in Figure 1. PZT transducers in the form of patches are adopted in this paper since they can be easily bonded on the out surface of interested structure. As shown in Figure 1, the PZT1 patch generates stress waves that propagate across the interface of the timber connection, and the received signal is captured by the PZT2 patch. Both patches are bonded on the top surfaces of the timber specimens. Due to the damage of timber connections, the actual contact area of structures will change and lead to a corresponding energy change of the stress wave (i.e., attenuation). The wavelet packet energy approach is used to quantify the signal energy attenuation, and thus the damage extent of timber connections can be detected.

### 2.2. Wavelet Packet Energy

The wavelet-packet approach has been widely used in analyzing signals, due to its satisfactory characteristics, such as high time-frequency resolution and effective decomposed capacity. In order to monitor the damage of different timber connections in real time, the wavelet packet analysis is applied in this paper to quantify the attenuation of signal energy at different frequency bands. Even though formulas of the wavelet packet energy analysis are very well-known, we still introduce their principle briefly in this section to clarify the procedure of establishing index, which is given as follow.

Firstly, by a n-level wavelet packet decomposition, the original signal S received by the PZT sensor is decomposed into $2^{n}$ signal subsets with different frequency bands. The signal subset $X_{j}$ can be expressed as [43],

(1) $$X_{j}=\left[X_{j,1},X_{j,2},\cdots ,X_{j,m}\right]$$

where m is the data sampling of the decomposed signal subset; j is the frequency band $\left(j=1,2,\ldots ,2^{n}\right)$.

Subsequently, the energy of the signal subset $E_{i,j}$ is defined as,

(2) $$E_{i,j}={\left\|X_{j}\right\|}^{2}=X_{{}_{j,1}}^{{}^{2}}+X_{{}_{j,2}}^{{}^{2}}+\cdots +X_{{}_{j,m}}^{{}^{2}}$$

where i was the ith measurement.

The energy vector for the signal at the ith measurement can be given as,

(3) $$E_{i}=\left[E_{i,1},E_{i,2},\cdots ,E_{i,2^{n}}\right]$$

Finally, based on the definition of the energy vector $E_{i}$, the total energy $E_{}$ of the received original signal at the ith measurement can be computed as,

(4) $$E=\sum_{j=1}^{2^{n}}E_{i,j}$$

## 3. Experimental Setup

**Figure 2:**
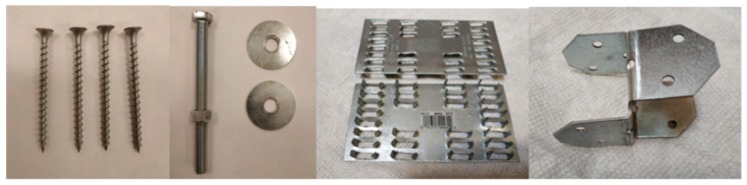
Different connection components.

The most used connection parts in timber constructions are mechanical fasteners, and four common types of timber connections are used in this paper, as shown in Figure 2.

### 3.1. Timber Specimen

**Figure 3:**
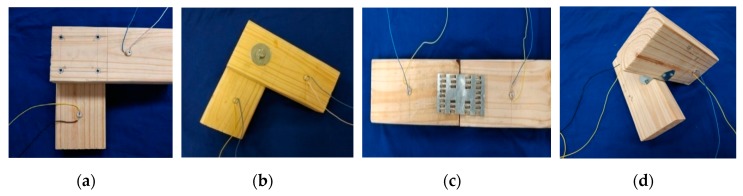
Timber specimens with different connections. (**a**) The screw connection; (**b**) the bolt connection; (**c**) the metal tooth plate connection; and (**d**) the decussation connection.

Four groups of timber specimens (pine wood from North America) with the same dimensions (200 mm × 40 mm × 90 mm) are used in this experiment. Each group has two pieces of the specimens fastened by one connection type, and thus there are a total of eight specimens, as shown in Figure 3: Group A is a connection with four screws (#6*3/4in); Group B is a bolt connection (HEX Bolt 3/8in); Group C is a metal tooth plate connection (MP24), and Group D is a decussation connection (RTB22). For each group, two PZT patches (ϕ 10 mm × 2mm), which are sandwiched structures with two electrode layers and one layer of PZT, were mounted at the predetermined location using epoxy (Loctite Heavy Duty 5 min epoxy).

Moreover, in this paper, the monitoring of timber connection damage based on active sensing method was performed with artificial damages. The practical damage levels are described as follows: (1) for Group A, Case1-A is the initial health status with four screws all tightened, then we damage the integrity by loosening one, two, and three screws, respectively. In accordance with the different damage levels, the damage cases are called Case2-A, Case3-A, Case4-A, respectively. (2) for Group B, a torque wrench is used to apply the pre-load from 5 to 20 N m. Case1-B is considered as the intact case with the maximal applied torque 20 N m, then the damage cases, namely Case2-B, Casee3-B, and Case4-B, are assigned as 15, 10, and 5 N m, respectively; (3) for Group C, two timber specimens are fastened by two metal tooth plates that have short and sharp nails, and the damage is simulated by prying up metal tooth plates under different levels. Specifically, Case1-C is considered as the intact case with two tightened tooth plates, Case2-C, Case3-C, and Case4-C are the damage cases with the tooth plates loose slightly, moderately and severely, respectively. (4) for Group D, two timber specimens are connected and fixed by a decussation part with screws, and we mimic different damage cases by loosening screws. Case1-D is considered as the intact case with the tightened decussation, then the damage cases, labeled as Case2-D, Case3-D, and Case4-D are damaged by loosening one, two, and three screws of the connection decussation, respectively. In order to describe the intact and damage cases more clearly, damages of timber connections in four groups are summarized in Table 1.

### 3.2. Experimental Setup and Experimental Procedure

**Figure 4:**
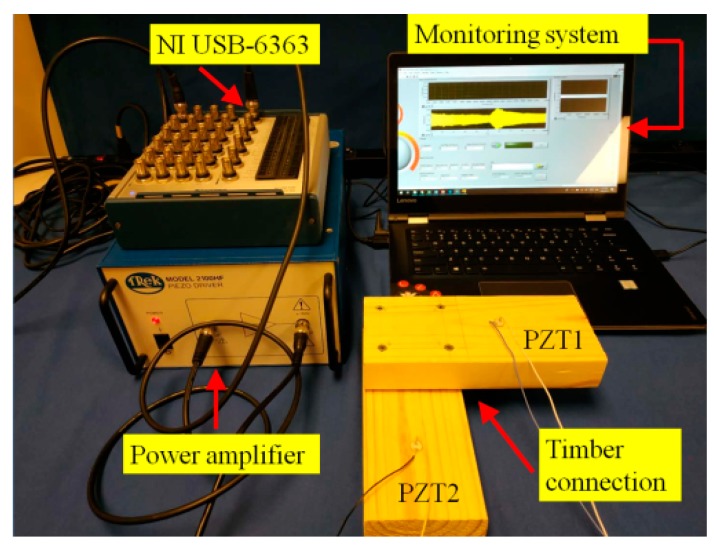
Experimental setup.

The experimental apparatus includes a data acquisition system (NI USB-6363), a signal power amplifier (Trek model 2100 HF), a laptop and timber connections of Group A, B, C, and D (as depicted in Figure 4).

The sampling frequency of the data acquisition system is 1 MHz. For each group, a swept sine wave signal (from 100 kHz to 300 kHz) with amplitude of 5 V was applied to excite PZT actuator, and the generated stress wave propagated from one timber to the other. The PZT sensor captured the response signal from the other timber. On the other hand, all tests in this manuscript were finished within two hours in the laboratory to minimize the influence of the environmental issues on the results, and thus the humidity and temperature change during the tests could be ignored. Moreover, we will conduct further investigations on the temperature effects on PZTs in the future work.

## 4. Results and Discussions

**Figure 5:**
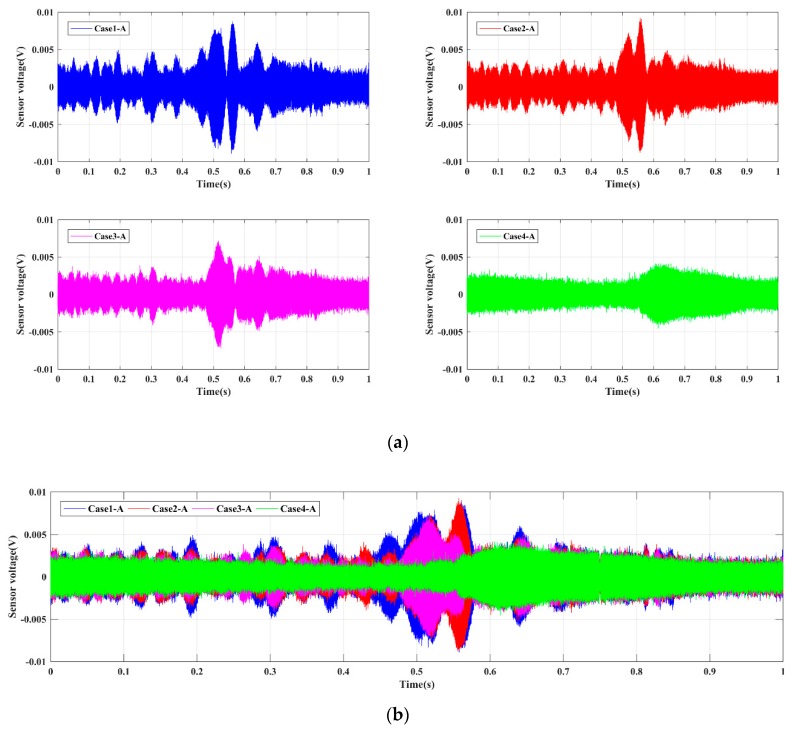
Sensor signal response for Group A. (**a**) Single case; (**b**) comparative cases.

**Figure 6:**
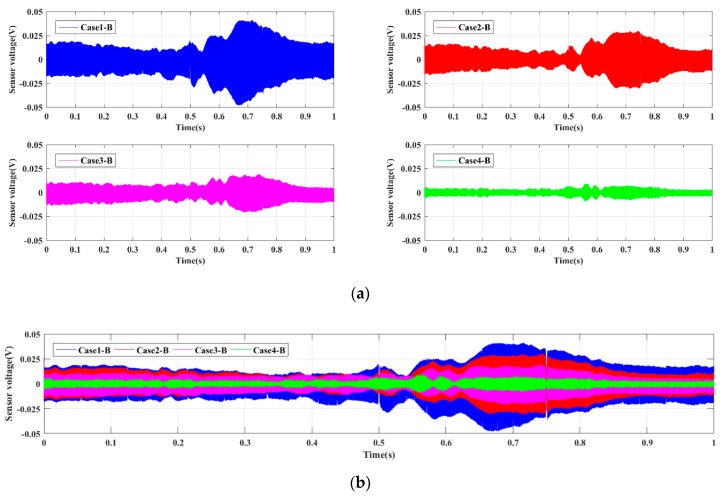
Sensor signal response for Group B. (**a**) Single case; (**b**) comparative cases.

**Figure 7:**
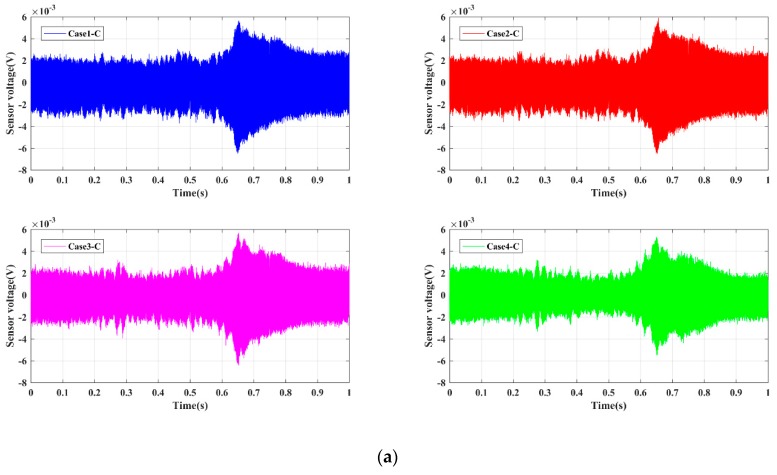

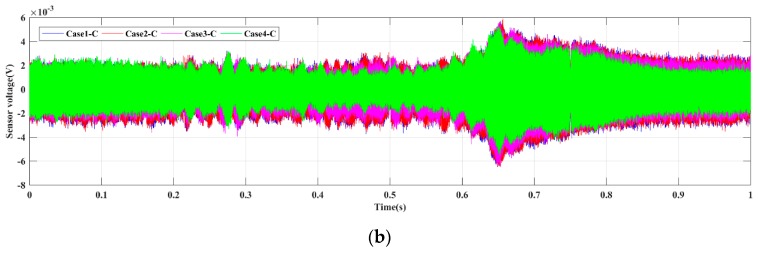
Sensor signal response for Group C. (**a**) Single case; (**b**) Comparative cases.

**Figure 8:**
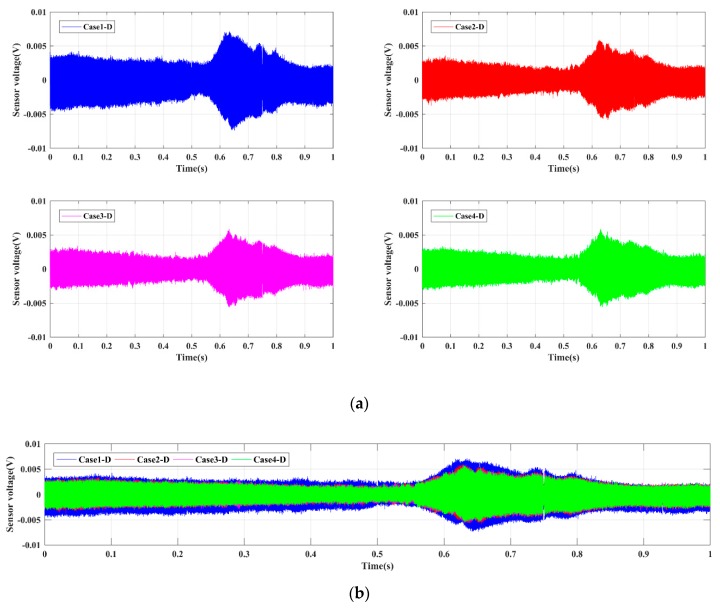
Sensor signal response for Group D. (**a**) Single case; (**b**) comparative cases.

For the four Groups A, B, C, and D, the time-domain signals received by PZT sensors are shown in Figure 5, Figure 6, Figure 7 and Figure 8, respectively. The results demonstrate that the amplitude of signals decrease with the increase of the damage. In other words, the intact status (Case1 in every group) has the largest amplitude, and amplitudes under the damage status (i.e., Case2, Case3, and Case4 in every group) decrease sequentially. Moreover, the Group B, namely the bolt connection, shows the most obvious tendency among four different groups, and the next was the Group A, i.e., the screw connection. The change of Group C (the metal tooth plate connection) and Group D (the decussation connection) are not as obvious as the first two. It is worth noting that the wave shapes under each case are different from each other. This phenomenon can be explained through the structural stiffness changes under different cases. We applied linear swept sine waves to excite the PZT patch, in other words, the time-domain waves also present frequency characteristics. It is well known that structural damages can induce corresponding stiffness changes, thus the wave shape under each case is different. The experimental results reveal that the active sensing method has potential to achieve real-time monitoring of the damage of timber connections.

**Figure 9:**
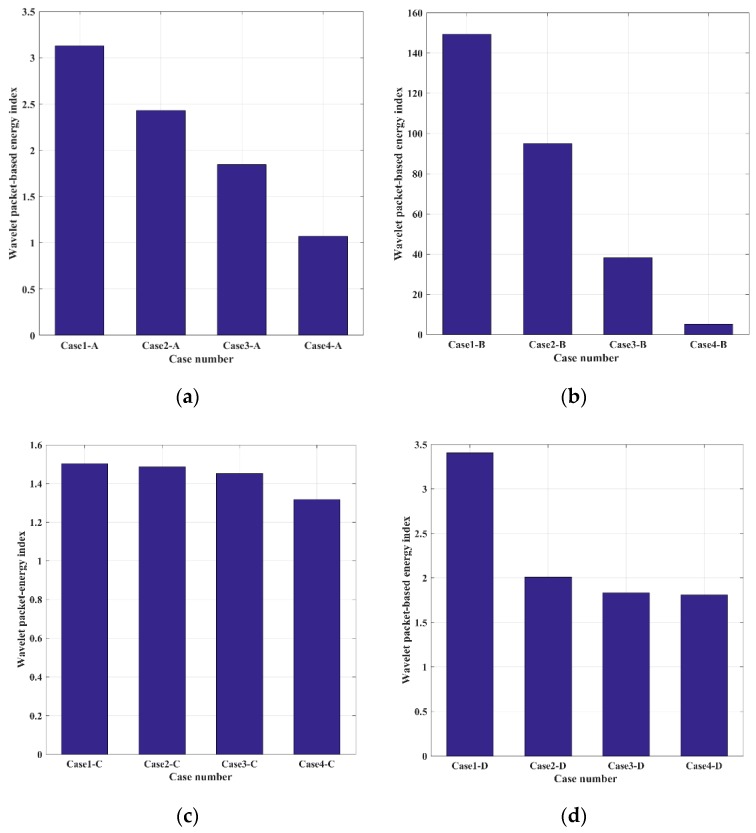
Energy indices for different timber connections in (**a**) Group A, (**b**) Group B, (**c**) Group C, and (**d**) Group D.

In order to quantify the received stress waves, the energy of the received signal was computed through the wavelet packet energy method, and the results are shown in Figure 9. It can be found that the energy of received signals in four groups decreased with the increase of damages of timber connections. Additionally, there are some differences among the four groups. For instance, the energy in Group A and B decreased dramatically the more severe the damages to the timber connections were. However, the decreasing trend in Group C was not obvious, and energy attenuation in Group D tended to saturate. These differences may be attributed to the different types of timber connections. It is well known that the PZT-enabled active sensing method depends on stress wave propagation, particularly, the energy attenuation when stress wave propagates through the interface. Generally, a larger interface area means that more stress wave energy can be transferred and received by the PZT patch. Thus, timber connections with bolts and screws, whose principle are similar, are more sensitive to active sensing, since preloads are proportional to interface areas in these two types of connections. On the other hand, the wave propagation paths of tooth plate connections and decussation joints are different. For example, the propagation paths of tooth plate connections during active sensing method are: PZT1→one timber specimen→teeth→plate→teeth→the other specimen→PZT2. Similarly, in the decussation joints, the stress wave will propagate across the paths: PZT1→one timber specimen→screws→decussation→screws→the other specimen→PZT2. We found that the loosening damages in these two connections (i.e., the debonding of teeth and screw looseness) have a smaller impact on wave propagation, since they affect the interface slightly. Therefore, the PZT-enabled active sensing has unsatisfactory performance in detecting the tooth plate connections and the decussation joints.

Overall, the experimental results demonstrated that the active sensing method has a great potential to monitor damages of timber connections. However, there are still many challenges in applying this technology for practical use. Firstly, the investigation in this paper considered the only four common connection types; other joints such as epoxy-bonded connection and mortise–tenon connection are required to be studied in future study. Additionally, some factors such as boundary conditions, the effect of wood type, and geometry size of the specimen were not considered. Future investigations will be conducted to address these issues.

## 5. Conclusions

Timber structures are widely used in industries, due to their merits such as being low-cost. The damage of timber connections directly affects the safety and reliability of timber structures. Thus, this paper aims to apply a stress wave-based active sensing approach to monitor damages of common timber connections. Four common types of timber connections (screwed connection, bolted connection, metal tooth plate, and decussation) were considered in this paper. Surface-bonded PZT patches were used in the active approach. The experimental results showed that the amplitude of received signals decreased with the increase of damage extent in timber connections, and the energy of the received signals, which was computed by the wavelet packet energy method, could be used to quantify the damage of the timber connections. Moreover, it is worth noting that the energy decreased dramatically with more severe damages when we detected screw connections and bolted connections in timber structures. However, the decreasing trend was not obvious during the detection of tooth plate connection, and energy attenuation for monitoring the decussation connection tended to saturate. The difference may be attributed to the different types of timber connections, and we will propose a more advanced method to solve this issue. Overall, we demonstrated that the active sensing method based on PZT transducers is effective and sensitive to monitor the damages of timber connections in real time. Therefore, regarding the widely-used wooden houses in America and Japan, we can apply this method to detect connection damages to ensure structural integrity and protect property.

Recently, vision-based structural health monitoring (SHM) methods, particularly for bolt-loosening detection [44,45], have been reported. However, there are several demerits of the current visual-based detection of bolt looseness, for instance, a loose bolt that has exactly one circle rotation cannot be detected. Therefore, our future work will focus on the improvement of the current vision-based methods for bolt loosening detection, to overcome existing problems.

## Figures and Tables

**Table 1:** Test cases of connection damage in Group A, B, C, and D.

Group Number	Intact Case	Damage Case		
Group A	Case1-A	Case2-A	Case3-A	Case4-A
	four tightened screws	loose one screw	loose two screws	loose three screws
Group B	Case1-B	Case2-B	Case3-B	Case4-B
	*T* = 20 N m	*T* = 15 N m	*T* = 10 N m	*T* = 5 N m
Group C	Case1-C	Case2-C	Case3-C	Case4-C
	two tightened plates	loose slightly	Loose moderately	loose severely
Group D	Case1-D	Case2-D	Case3-D	Case4-D
	tightened decussation	loose one screw	loose two screws	loose three screws
